# Subtype-specific alternative splicing events in breast cancer identified by large-scale data analysis

**DOI:** 10.1038/s41598-024-65035-y

**Published:** 2024-06-19

**Authors:** Yui Deguchi, Chie Kikutake, Mikita Suyama

**Affiliations:** Division of Bioinformatics, Medical Institute of Bioregulation, Kyushu University, Fukuoka, 812-8582 Japan

**Keywords:** Cancer genomics, RNA splicing

## Abstract

Genome analysis in cancer has focused mainly on elucidating the function and regulatory mechanisms of genes that exhibit differential expression or mutation in cancer samples compared to normal samples. Recently, transcriptome analysis revealed that abnormal splicing events in cancer samples could contribute to cancer pathogenesis. Moreover, splicing variants in cancer reportedly generate diverse cancer antigens. Although abnormal splicing events are expected to be potential targets in cancer immunotherapy, the exploration of such targets and their biological significance in cancer have not been fully understood. In this study, to explore subtype-specific alternative splicing events, we conducted a comprehensive analysis of splicing events for each breast cancer subtype using large-scale splicing data derived from The Cancer Genome Atlas and found subtype-specific alternative splicing patterns. Analyses indicated that genes that produce subtype-specific alternative splicing events are potential novel targets for immunotherapy against breast cancer. The subtype-specific alternative splicing events identified in this study, which were not identified by mutation or differential expression analysis, bring new significance to previously overlooked splicing events.

## Introduction

Cancer genome analysis has focused mainly on understanding the functions and regulatory mechanisms of genes that exhibit differential expression or mutations in cancer samples compared to normal ones^1^. Recently, transcriptome analysis has reported cases in which splicing variants that alter protein-coding sequences in cancer affect tumor cell growth. For example, the variant of the U2AF1 splicing factor induces abnormal splicing events in lung adenocarcinoma and myelodysplastic syndrome by recognizing a different sequence as the 3' splice site than normal^2–4^. Furthermore, the SF3B1 variant recognizes a different branch point, altering splicing patterns in uveal melanoma and lymphocytic leukemia^5,6^. Splicing variants in cancer have also been reported to generate various cancer antigens^7,8^, which play a crucial role in predicting the prognosis of various types of cancer, including breast and gastric cancer^9,10^.

Since the introduction of the immune checkpoint inhibitor, Nivolumab, which activates T cell responses against cancer cells by binding to PD-1 on T cells, immunotherapy has become a prominent cancer treatment^11–14^. One type of immunotherapy is peptide vaccine therapy, in which the patient is inoculated with an antigen (neoepitope) as a vaccine, which is a peptide derived from the mutation, to activate the patient’s T cells, thereby inducing a strong immune response and suppressing cell growth. Recently, it has been suggested that cancer-specific peptides resulting from alternative splicing (AS) as well as those resulting from mutations can also contribute to immunogenicity regulation by functioning as neoantigens^7,15–17^. However, due to the complexity of the regulatory mechanism of AS, cancer immunotherapy target exploration based on AS events and their biological significance has not been completely elucidated. Furthermore, although most AS analyses have been performed for each type of cancer, the subtypes have not been adequately analyzed for more effective therapies.

In this study, we focus on breast cancer (BRCA), which is characterized by multiple subtypes that require optimal subtype-specific cancer treatment. Using AS event data from 1,079 patients with BRCA obtained from The Cancer Genome Atlas (TCGA)^7,18,19^, we conducted a comprehensive analysis of AS and identified 660 genes showing subtype-specific AS events in BRCA. These genes represent potential novel targets for immunotherapy against BRCA. In this study, splicing events not identified by mutation or differential expression analysis, provide new knowledge on splicing events that have been overlooked.

## Materials and methods

### Datasets

We obtained Percent Spliced In (PSI) values data from tumor samples derived from 32 types of cancer types (*n* = 9349) and normal samples obtained from the same patients (*n* = 670) (https://gdc.cancer.gov/about-data/publications/PanCanAtlas-Splicing-2018)^7,20^ from TCGA. The data have PSI values for five types of AS events: Exon skipping (ES), alternative 5′ sites (A5), alternative 3′ sites (A3), mutually exclusive exons (MX), and intron retention (IR). From this data, we extracted tumor sample (*n* = 1088) and normal sample (*n* = 110) data for BRCA. The five BRCA subtypes [Basal-like (Basal), Luminal A (LumA), Luminal B (LumB), HER2-enriched (Her2), and Normal Breast-like] information was obtained from the cBioPortal for Cancer Genomics (cBioPortal) (https://www.cbioportal.org/study/clinicalData?id=brca_tcga_pan_can_atlas_2018)^21^. Furthermore, we retrieved gene expression data of TCGA-BRCA samples from the Genomic Data Commons Data Portal (GDC Data Portal) (https://portal.gdc.cancer.gov) as data calculated in transcripts per million (TPM) and clinical data from https://gdc.cancer.gov/about-data/publications/pancanatlas. After downloading The Matched Annotation from NCBI and EMBL-EBI (MANE) select (https://ftp.ncbi.nlm.nih.gov/refseq/MANE/MANE_human/release_1.1/MANE.GRCh38.v1.1.ensembl_genomic.gff.gz)^22^ for GRCh38 from UCSC, the human genome reference sequences were converted to coordinates corresponding to GRCh19.v1.1 using liftOver (v.1.04.00). Databases used for gene classification included the oncogene database (https://ongene.bioinfo-minzhao.org/search_result.cgi), TUMOR SUPPRESSOR GENE DATABASE (https://bioinfo.uth.edu/TSGene/index.html?csrt=15727984646926095482), JCGA (https://www.jcga-scc.jp/ja), COSMIC (https://cancer.sanger.ac.uk/cosmic), AS-CMC^23^ (https://www.pmrc.re.kr/ASCMC/), and THE HUMAN PROTEIN ATLAS (HPA).

(https://www.proteinatlas.org/).

### Filtering and processing of the AS event data

AS events PSI = 0.0 (complete exclusion of the exon) or PSI = 1.0 (complete inclusion of the exon) in all samples were removed from the AS event data for the BRCA samples (filtering criteria [1])^7^. Next, if the number of PSI values with NaN (missing values) was present in > one-tenth of the total number of BRCA samples, the corresponding AS event was removed (filtering criteria [2]). This cutoff value was used according to the previous study^7^. Furthermore, if no subtype information was available, the sample was labeled as ”NA” and normal samples were labeled as “Normal tissue (normal)”^7^.

### Identification of genes showing subtype-specific AS events

To extract AS events that show specifically in each subtype, we divided the samples into two groups: samples corresponding to one subtype and a group of other subtypes. The Wilcoxon rank sum test was used to compare PSI values and expression levels between the two groups. Then, the obtained *P*-values were corrected using the Benjamini & Hochberg method (*q*-values)^24^. Three criteria for subtype-specific AS event extraction (Basal, LumA, LumB, and Her2) were as follows: (1) PSI must show a significant difference between the two groups (*q* < 0.05), (2) the expression levels (TPM) of genes showing AS events should not show a significant difference between the two groups (*q* > 0.05), and 3) the number of mutations at the splice site of genes showing AS events should not exhibit a significant difference between the two groups (*q* > 0.05).

### Protein structure analysis of subtype-specific AS regions

We obtained the amino acid sequence of the gene that shows subtype-specific AS events using the following methods. If the number of bases in the mRNA of the spliced region was a multiple of three and the normal amino acid sequences were not misaligned during splicing, we acquired the amino acid sequences from the gene revealing the subtype-specific AS region using UCSC. If the number of bases in the mRNA of the spliced region was a multiple of three but the amino acid sequence of the normal case was misaligned during splicing, we obtained the mRNA sequence from the gene demonstrating that the subtype-specific AS region using UCSC. Subsequently, we converted the mRNA sequence to an amino acid sequence using Python. The Alpha Fold Protein Structure Database (https://alphafold.ebi.ac.uk/) was used to determine the position of these amino acid sequences in the protein structure, and among them, we extracted those with splicing regions on the surface of the protein. For amino acid sequences with < 800 residues, we used the Google Colab version of AlphaFold2 (v.1.5.2) to predict protein structure^25^. For amino acid sequences exceeding 800 residues, we used LocalColabFold (v.1.5.0) to locally predict the protein 3D structure^26^. Finally, we compared the protein structure of a gene when a subtype-specific AS event occurred with the protein structure of a gene when no subtype-specific AS event occurred using PyMOL (v.1.20)^27^ and Coot for Windows (v.0.9.8.7.1)^28^.

### Survival analysis using PSI values

Cox proportional hazards model in the R survival package (v.3.5–7) was used to estimate hazard ratios (HRs) and their 95% confidence intervals of PSI values. We used cancer age at diagnosis, the AJCC pathologic stage, and subtype as covariates. The selection of these covariates was based on previous studies^29,30^.

### Statistical analysis

Statistical analyses were performed using R software version 4.0.1 (R Project for Statistical Computing, Vienna, Austria) and Python software version 3.8.3.

## Results

### Characteristics of AS events in the BRCA

To examine the characteristics of AS events in BRCA, we obtained data on AS events derived from tumor samples (*n* = 1,088) and normal samples (*n* = 110) in BRCA provided by TCGA. The total number of AS events in these samples was 1,723,249 (Tables 1 and 2).

**Table 1:** The number of samples of breast cancer subtypes.

Breast cancer subtype	Sample size
Basal-like(Basal)	175
Luminal A(LumA)	502
Luminal B(LumB)	196
HER2-enriched(Her2)	78
Breast-like(Normal)	36
Subtype unknown (NA)	101
normal sample (normal)	110
Sum	1198

**Table 2:** The number of five types of AS events in breast cancer.

Alternative splicing	Number of AS events	Filtering criteria [1]	Filtering criteria [2]
ES	339,859	195,314	52,702
A5	137,362	98,891	33,752
A3	181,915	135,646	53,112
IR	123,391	105,431	36,642
MX	940,722	836,714	8592
Sum	1,723,249	1,371,996	184,800

Next, we applied filtering criteria to exclude AS events with limited variation in PSI values between BRCA samples (filtering criteria [1] and [2] in “Filtering and processing of the AS event data” in the Materials and Methods section). The total number of selected AS events was 184,800 (Table 2). To characterize the PSI values associated with these splicing events, we calculated the mean PSI for each AS event (ES, A5, A3, MX, IR) (Supplementary Fig. S1). We used mean value instead of variance to evaluate the degree of exon skipping. Across all five AS events, the mean PSI values were highly skewed toward 1 and 0 (Supplementary Table S1). The frequency decreased as the mean PSI values deviated from 0 and 1. However, for MX, there was a notable variation in the distribution between 0.1 and 0.9, possibly due to uncertainty in the definition of MX.

### AS patterns in BRCA subtypes

**Figure 1:**
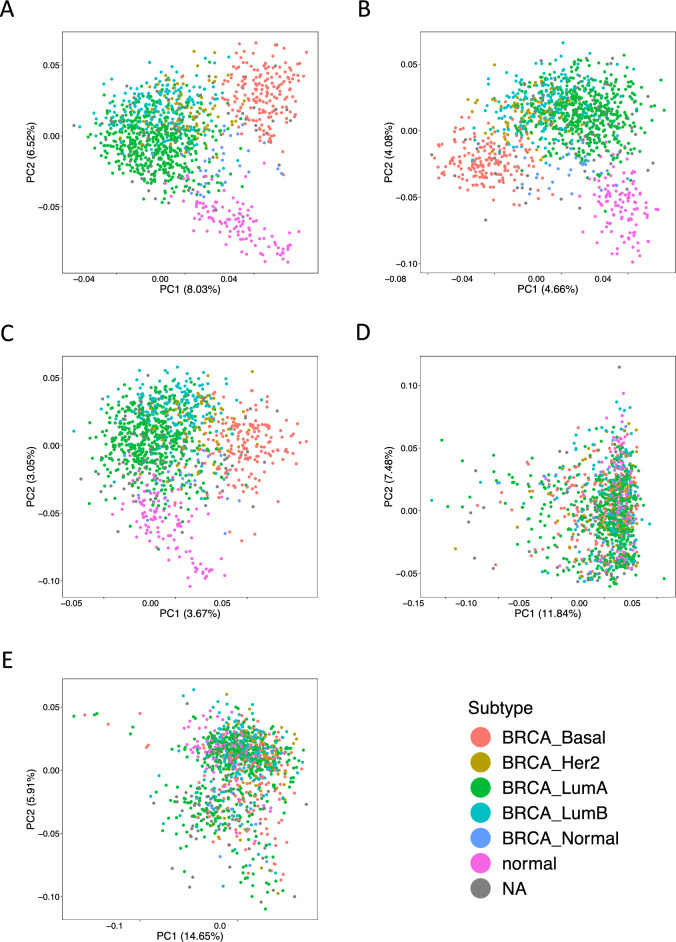
PCA results for subtype-specific AS patterns with mean PSI values of 0.3–0.7. PC1 and PC2 were calculated by PCA of PSI values for each AS event in BRCA samples. (**A**) PC1 and PC2 for ES, (**B**) A5, (**C**) A3, (**D**) MX, and (**E**) IR. Data from AS events with mean PSI values of 0.3–0.7 were used. The horizontal axis represents PC1 and the vertical axis represents PC2. The percentages on the horizontal and vertical axes represent the contribution ratio of PCA.

To investigate the distinctive characteristics of the AS patterns in each BRCA subtype, we conducted principal component analysis (PCA) using PSI values associated with AS events for each subtype (Fig. 1). Across five types of AS (ES, A5, A3, MX, and IR), we extracted AS events with mean PSI values of 0.0–1.0 (without cutoff for mean PSI values), 0.1–0.9, 0.2–0.8, 0.3–0.7, and 0.4–0.6 and applied PCA using the PSI values. No subtype-specific clusters were observed when using all AS event data (Supplementary Fig. S2). Using AS events with mean PSI values of 0.1–0.9 and 0.2–0.8 revealed some separation in subtype-specific groups for ES, A5, and A3 (Supplementary Figs. S3 and S4). In AS events with mean PSI values of 0.3–0.7, and 0.4–0.6, we observed distinctly separated clusters for each of the four subtypes (Basal, LumA, LumB, and Her2), particularly in ES, A5, and A3 (Fig. 1 and Supplementary Fig. S5). Normal and tumor samples were also separated into different clusters (Fig. 1 and Supplementary Fig. S5). These findings suggest that AS events with high PSI variation may exhibit specific AS patterns for each BRCA subtype. In subsequent analyses, we focused on three types of AS (ES, A5, and A3) in these four subtypes (Basal, LumA, LumB, and Her2).

### Exploration of genes showing subtype-specific AS events in BRCA

To examine the characteristics of genes showing subtype-specific AS events, we obtained subtype-specific AS events for each of the four subtypes using the three filtering criteria outlined in “Identification of genes showing subtype-specific AS events” in the Materials and Methods section (Table 2). Then we identified 660 genes associated with the 1,867 splicing events and found that, for each AS type (ES, A5, and A3), the number of genes associated with the Basal subtype dominates compared with the other subtypes (Table 3). Among these 660 genes, 351 genes (53%) had only one subtype-specific AS event, while the highest number of subtype-specific AS events (12 events) was observed in the mitochondrial fission factor gene (Supplementary Tables S2–S13). These 660 genes were classified into three categories using multiple databases: cancer-related genes, BRCA-related genes, and non-cancer genes. Notably, BRCA-related genes constitute a subset of cancer-related genes. We found that 573 genes (86.8% of the total genes) were cancer-related or BRCA-related (Table 3).

**Table 3:** The number of AS events and genes identified for each subtype.

	Subtype	Number of AS events	Number of genes	Number of cancer-related genes	Number of BRCA-related genes	Number of non-cancer genes
ES	Basal	707	358	313	80	45
	LumA	358	232	200	52	32
	LumB	176	127	116	35	11
	Her2	73	62	54	17	8
A5	Basal	147	106	95	27	11
	LumA	73	62	50	19	12
	LumB	59	48	42	14	6
	Her2	47	43	33	9	10
A3	Basal	127	98	89	24	9
	LumA	53	43	37	12	6
	LumB	29	25	22	3	3
	Her2	18	18	13	4	5
Total unique genes			660	573	162	87

**Figure 2:**
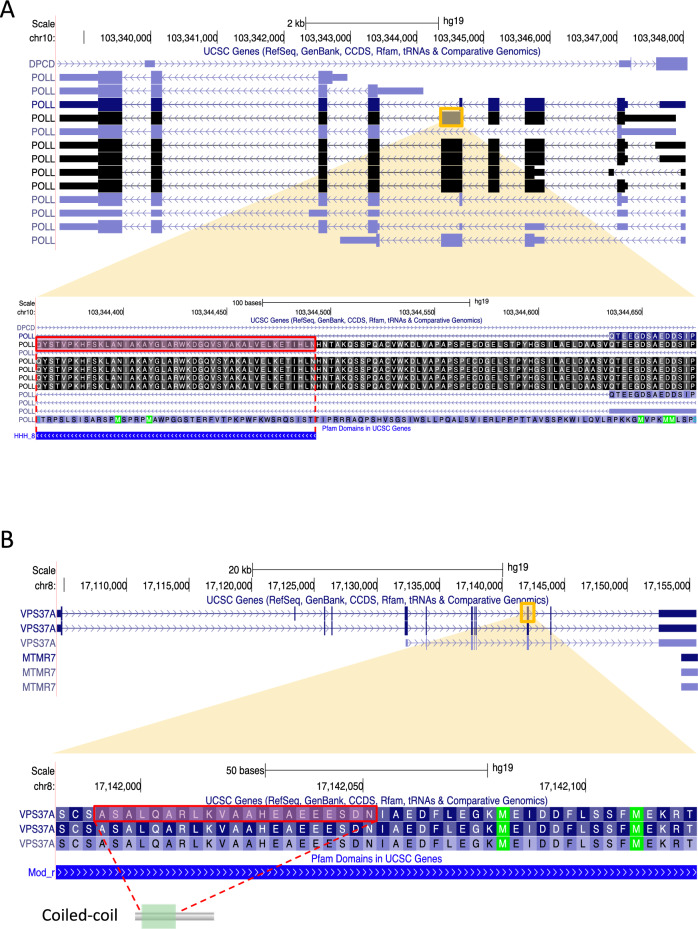
The potential function of AS regions in *POLL* and *VPS37A*. (**A**) Complete genomic structure of *POLL* The region shown in the yellow square is the Basal-specific AS region extracted in this analysis. The region highlighted in a pink square in the enlarged region overlaps with that of the Helix-hairpin-helix domain (HhH_8). (**B**) Complete genomic structure of *VPS37A* The region shown in the yellow square is the Basal-specific AS region extracted in this analysis. The region highlighted in a pink square in the enlarged region overlaps with the coiled-coil region.

To explore candidate cancer genes and their AS events in BRCA, we focused on 87 genes classified as non-cancer genes. We used HMMER (https://www.ebi.ac.uk/Tools/hmmer/) to examine the relationship between protein domains and regions associated with subtype-specific AS events in these genes. Our results revealed that 39.1% (34/87) of these genes exhibited overlap between subtype-specific AS events and biologically important regions, such as the helix-hairpin-helix motif (HhH), which is considered a DNA binding site, and the coiled-coil structure, which is involved in oligomer formation (Supplementary Table S14). For example, in the DNA Polymerase Lambda (*POLL*) gene, where Basal-specific AS occurs, the Basal-specific AS region overlapped with the region corresponding to the HhH motif (Fig. 2A). Similarly, in the Vacuolar protein sorting 37 homolog A (*VPS37A*) gene the Basal-specific AS region overlapped with the region producing the coiled-coil structure (Fig. 2B).

### Protein conformational changes caused by subtype-specific AS events

To explore AS events that may yield potential novel cancer markers or treatment targets for each BRCA subtype, we extracted subtype-specific AS events that met the following three conditions: (1) the sequence of the region of the AS event must be in-frame, (2) the region must reside on the surface of a protein structure, and (3) the protein structure prediction accuracy score (pLDDT) of the region predicted by AlphaFold2 must be ≥ 70. Applying these conditions, we identified 40 genes showing 33, 13, 8, and 3 (57 in total) subtype-specific AS events in Basal, LumA, LumB, and Her2 (Table 4). Among the genes, we focused on two genes that show Basal-specific AS events, glutathione S-transferase zeta 1 (*GSTZ1*) and myosin light chain 6 (*MYL6*).

**Table 4:** The number of candidate AS events and their genes.

	Subtype	Number of AS events	Number of out-flame AS events	Number of in-flame AS events	Number of candidate AS events	Number of candidate genes
ES	Basal	91	53	38	23	17
	LumA	50	31	19	10	9
	LumB	20	11	9	5	4
	Her2	7	4	3	0	0
A5	Basal	16	11	5	4	4
	LumA	13	9	4	2	2
	LumB	7	4	3	3	3
	Her2	10	7	3	3	3
A3	Basal	10	3	7	6	6
	LumA	7	6	1	1	1
	LumB	4	4	0	0	0
	Her2	5	5	0	0	0
Sum		240	148	92	57	40 (Total unique genes)

**Figure 3:**
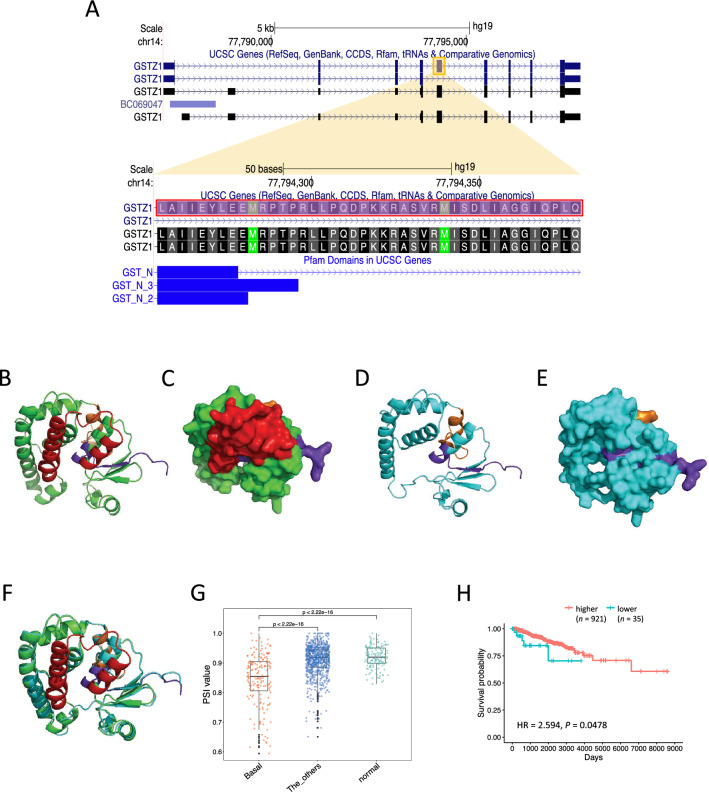
Predicted protein structure of GSTZ1 showing Basal-specific AS events. (**A**) Complete genomic structure of *GSTZ1*. The region shown in the yellow square is the Basal-specific AS region extracted in this analysis. The AS region is highlighted by a pink square in the enlarged region. (**B**) Predicted protein structure of GSTZ1 without Basal-specific exon skipping. The exon skipping region is colored red. The purple region is the N-terminal region. The orange region is the C-terminal region. (**C**) Surface display of the predicted protein structure of GSTZ1 without the Basal-specific exon skipping by PyMOL. (**D**) Predicted protein structure of GSTZ1 with Basal-specific exon skipping. (**E**) Surface display of the predicted protein structure of GSTZ1 with Basal-specific exon skipping by PyMOL. (**F**) Superposition of the predicted protein structure in (**B**) and (**C**). (**G**) Box plot showing PSI values of the Basal-specific AS events showing in *GSTZ1* for each of the three subtype groups. The vertical axis shows the PSI values, and the horizontal axis represents the subtypes. “The_others” represents the three subtypes other than Basal (LumA, LumB, and Her2). (**H**) Kaplan–Meier survival curves of breast cancer patients and their PSI values of the Basal-specific AS events showing in *GSTZ1*. The horizontal and vertical axes represent overall survival (days) and probability, respectively.

Among the genes with extracted splicing events, *GSTZ1* showed exon skipping of the Basal-specific AS event (Fig. 3A). When superimposing the three-dimensional structures of the protein (Fig. 3B,C), no significant differences in the protein’s orientation of the protein were observed (Fig. 3D–F). We found that PSI values with Basal-specific AS events were significantly lower in Basal compared to other groups (Fig. 3G). To examine the effect on patient prognosis, all breast cancer samples without missing values in clinical data were divided into two groups: one with extremely low PSI values (lower 5%: *n* = 35; Basal: *n* = 23, others: *n* = 12) and the other with higher PSI groups (*n* = 921; Basal: *n* = 143, others: *n* = 778). Our results showed that samples with lower PSI values were associated with a significantly poor OS (overall survival) time (HR = 2.594, *P* = 0.0478) (Fig. 3H and Supplementary Table S15). However, there was no significant difference in PFS (progression-free survival) time (HR = 1.805, *P* = 0.170).

**Figure 4:**
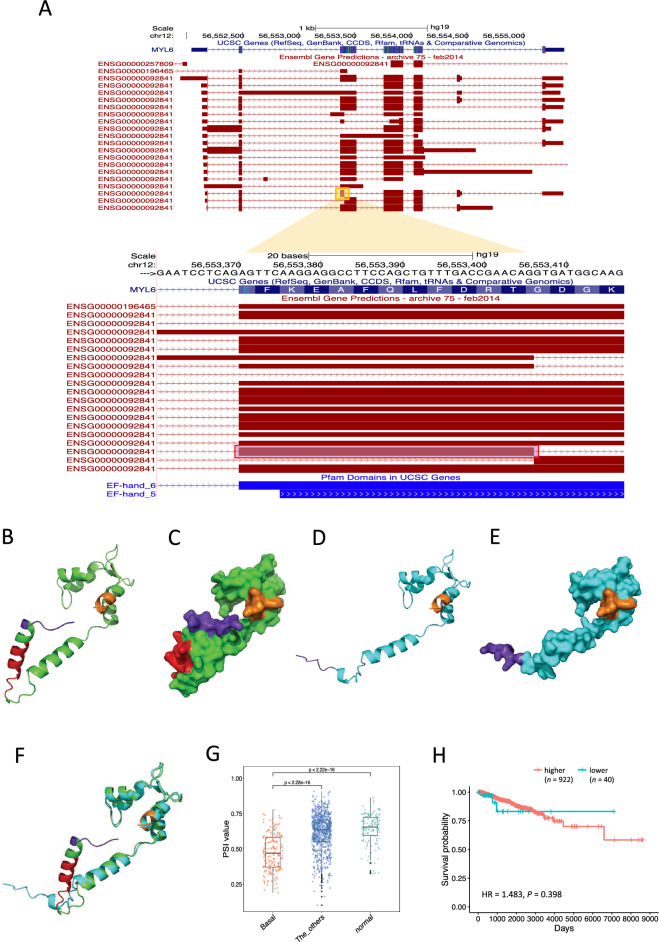
Predicted protein structure of MYL6 showing Basal-specific AS events. (**A**) Entire genomic structure of *MYL6*. The region shown in the yellow square is the Basal-specific AS region extracted in this analysis. The AS region is highlighted in a pink square in the enlarged region. (**B**) Predicted protein structure of MYL6 without Basal-specific exon skipping. The exon skipping region is colored red. The purple region is the N-terminal region. The orange region is the C-terminal region. (**C**) Surface display of the predicted protein structure of MYL6 without Basal-specific exon skipping by PyMOL. (**D**) Predicted protein structure of MYL6 with Basal-specific exon skipping. (**E**) Surface display of the predicted protein structure of MYL6 with Basal-specific exon skipping by PyMOL. (**F**) Superposition of the predicted protein structure in (**B**) and (**C**). (**G**) Boxplot showing PSI values of the Basal-specific AS events showing in *MYL6* for each of the three subtype groups. The vertical axis shows the PSI values, and the horizontal axis represents the subtypes. “The_others” represents the three subtypes other than the Basal subtype (LumA, LumB, and Her2). (**H**) Kaplan–Meier survival curves of breast cancer patients and their PSI values of the Basal-specific AS events showing in *MYL6*. The horizontal and vertical axes represent overall survival (days) and probability, respectively.

As another example, we show the event found in *MYL6* (Fig. 4A), which shows that exon skipping of the Basal-specific AS region in MYL6 alters the protein structure. To examine the impact of this AS event on proteins, we compared the three-dimensional structures of MYL6 using AlphaFold2. Notably, the structures differed significantly between proteins with and without Basal-specific AS. To further clarify the difference in the predicted protein structure with and without exon skipping, the three-dimensional structures of the protein were superimposed (Fig. 4B,C). In the case of the superposition of the C-terminal region, the orientation of the structure was altered in the middle portions of the protein (Fig. 4D–F). We also found that PSI values with Basal-specific AS events were significantly lower in Basal groups compared to other groups (Fig. 4G). As previously described to perform survival analysis to examine the effect on patient prognosis, all breast cancer samples without missing values in clinical data were divided into two groups: one with extremely low PSI values (lower 5%: *n* = 40; Basal: *n* = 28, others: *n* = 12) and the other with higher PSI values (*n* = 922; Basal: *n* = 142, others: *n* = 780). Survival analysis demonstrated no difference between samples with higher and lower PSI values in OS time (HR = 1.483, *P* = 0.398) (Fig. 4H and Supplementary Table S16) and PFS time (HR = 1.233, *P* = 0.647).

## Discussion

Previous studies revealed the existence of cancer-type-specific AS patterns^7^. These findings suggest that AS can help elucidate the causes of cancer and develop cancer immunotherapy. To explore subtype-specific AS events, we focused on the BRCA subtypes, and explored AS events specific to each subtype using AS event data provided by TCGA. We identified 1,867 subtype-specific AS events in 660 genes, 40 of which were non-cancer genes. Some of these events correspond to regions on the surface of the protein structure, and it was predicted that the protein structure would significantly alter due to the AS events. Some of the peptides resulting from these AS events can function in cancer cells as cancer-specific antigens called neoepitopes^31^. When the immune system recognizes these neoepitopes, it triggers an immune response that specifically attacks cancer cells, leading to suppressing their proliferation^32^. Therefore, the subtype-specific AS events identified in this study can provide valuable insights for effective cancer immunotherapy.

Among the four types of subtype-specific (Basal, LumA, LumB, and Her2) AS events regarding ES, A5, and A3, we observed the highest number of Basal-specific AS events. This outcome is partly because the Basal subtype has distinctive characteristics compared to the other three subtypes (LumA, LumB, and Her2). It is a triple-negative type that lacks expression of the estrogen receptor, the progesterone receptor, or HER2^33^. Furthermore, there are currently no effective therapeutic agents or treatments for the Basal subtype^34^, resulting in a higher recurrence rate after surgery compared to other subtypes^35^. Our findings suggest that Basal-specific AS events could serve as novel therapeutic targets for Basal subtype cancers.

In this study, we found that the Basal-specific AS region in *POLL* overlaps with the region corresponding to the HhH motif. *POLL* encodes a DNA polymerase that catalyzes the elongation of the 3' end of a DNA strand using a DNA template^36^. It reportedly plays a crucial role in nonhomologous end joining and other DNA repair processes^36^. Furthermore, *POLL* expresses transcript variants due to AS events^37^. The transcript variants derived from Basal-specific AS may induce abnormalities in the DNA repair process, and potentially lead to cancer. Also, we observed that the Basal-specific AS region that shows in *VPS37A* overlaps with the region corresponding to the coiled-coil structure. VPS37A, a member of the VPS37 family, encodes a component of the ESCRT-I (endosomal sorting complex required for the transport I) protein complex, which is essential for the sorting of ubiquitinated transmembrane proteins into internal vesicles of multivesicular bodies^38,39^. Numerous variants of the transcript due to AS events have also been reported for this gene^40^. Due to Basal-specific AS in *VPS37A*, the normal synthesis of components belonging to the ESCRT-I protein complex can be disrupted. This disruption may prevent the normal classification of ubiquitinated transmembrane proteins into the internal vesicles of multivesicular bodies, leading to abnormal signal transduction and cellular responses^40^. This, in turn, has the potential to contribute to the development of cancer and other diseases.

In a comparative analysis of the protein structure of AS regions, we focused on *MYL6*, which undergoes specific AS events in the Basal subtype. *MYL6* encodes both smooth muscle and myosin light chain isoforms^41,42^. The Basal-specific AS region in *MYL6* overlaps with the EF-hand motif^43–45^, which buffers intracellular calcium levels and confers various structural and functional diversity in proteins. Notably, the AS of this gene that affects cancer has not been reported. Therefore, the AS events identified in this gene may be a novel cancer-associated factor. In the breast, which is supported by the major pectoralis muscle, muscle-related MYL6 can affect the muscles near the breast through Basal-specific AS events.

AS may be directly involved in carcinogenesis but also be associated with drug resistance in cancer^46^. A mechanism for such resistance is the alteration of drug metabolism by AS. For example, AS-induced inactive forms of dCK are responsible for the loss of sensitivity to the anticancer drug cytarabine^47^. *GSTZ1*, which exhibits subtype-specific AS, as shown in the present study, could be one such example. The expression level of GSTZ1 is associated with susceptibility to dichloroacetate (DCA), a possible anti-cancer drug^48^. Identification of the subtype-specific AS event in this gene suggests the existence of a subtype-specific susceptibility to DCA in BRCA, even in the absence of differential expression levels.

This study has several limitations. Although we observed a significant difference in PSI values for extracted subtype-specific AS events, the magnitude of the difference was generally small in most cases. Therefore, a comprehensive analysis is crucial for understanding the impact of AS events on cancer, considering clinical data such as patient survival time and age. Furthermore, due to the absence of available proteomic data, we were unable to analyze the quantity of proteins translated from genes that show subtype-specific AS events. The availability of proteome data corresponding to AS events identified in this study could facilitate the estimation of the effects of translation products on cancer cells.

Among subtype-specific AS events, numerous genes were not associated with cancer pathogenesis. This finding highlights the importance of paying attention to AS events. By elucidating AS patterns for each subtype, in the specific BRCA subtype examined in this study and the other 31 species provided by TCGA, novel targets can be identified to develop more effective subtype-specific treatments.

### Supplementary Information

Supplementary Figures.

Supplementary Tables.

## Data Availability

All datasets are freely available from public databases. The results shown here are mainly based on data generated by TCGA: https://gdc.cancer.gov/about-data/publications/PanCanAtlas-Splicing-2018 and https://portal.gdc.cancer.gov. We also used subtype information from cBioPortal: https://www.cbioportal.org/study/clinicalData?id=brca_tcga_pan_can_atlas_2018, human genome reference sequences from MANE select: https://ftp.ncbi.nlm.nih.gov/refseq/MANE/MANE_human/release_1.1/MANE.GRCh38.v1.1.ensembl_genomic.gff.gz. The datasets and scripts generated in the present study are available from the corresponding author upon reasonable request. Further information and requests for codes and scripts generated in the present study should be directed to and will be fulfilled by the Lead Contact, Mikita Suyama (mikita@bioreg.kyushu-u.ac.jp).
