# Supplementary material for: Subtype-specific alternative splicing events in breast cancer identified by large-scale data analysis
Source: Sci Rep. 2024 Jun 19;14:14158. doi: 10.1038/s41598-024-65035-y (PMC11187070; doi:10.1038/s41598-024-65035-y)
Supplement: Supplementary file 1 — Supplementary Figures. [file 41598_2024_65035_MOESM1_ESM.pdf]

Fig. S1
Y Deguchi

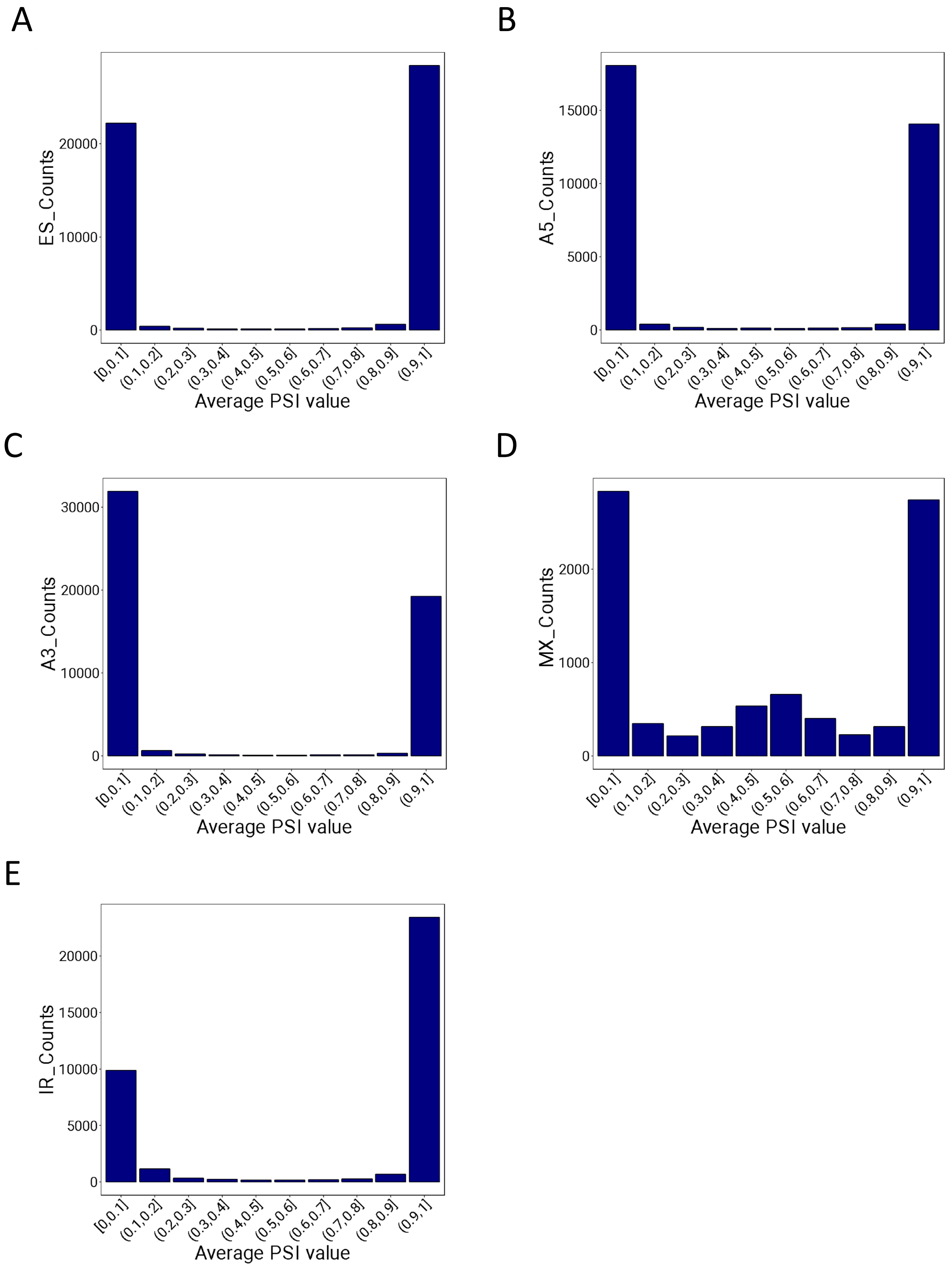

Supplementary Figure. S1 Characteristics of five types of AS events in BRCA

The distribution of PSI values for each AS events in BRCA samples. (A) PSI values for ES, (B) A5, (C) A3, (D) MX, and (E) IR. The vertical axis represents the number of AS events. The horizontal axis represents the average value of PSI. The maximum value on the horizontal axis is 1.0, the minimum value is 0.0, and the bin size is 0.1.

Fig. S2
Y Deguchi

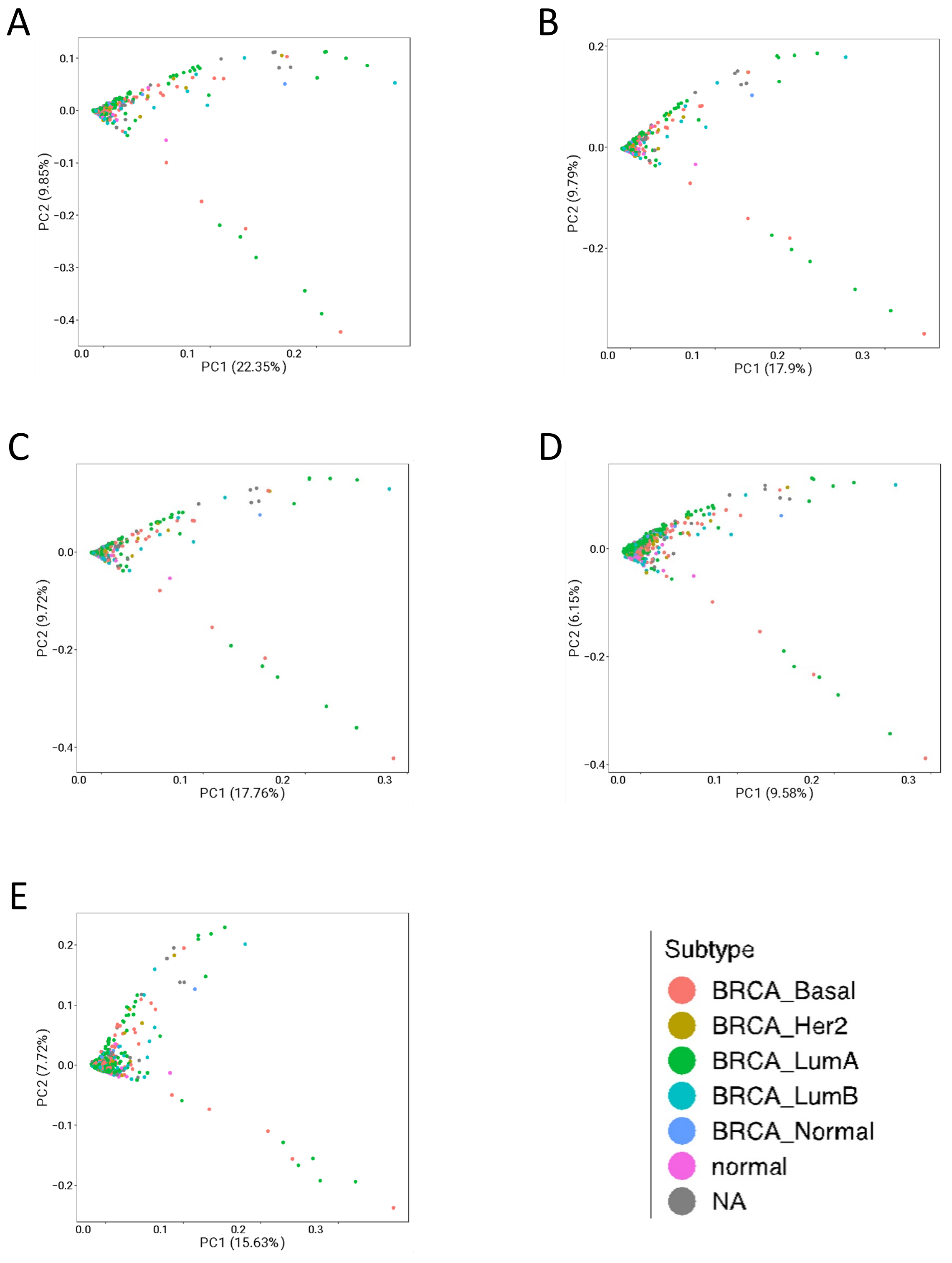

Supplementary Figure. S2 PCA results for subtype-specific AS patterns with average PSI values of 0.0–1.0

PC1 and PC2 calculated by PCA of PSI values for AS events in breast cancer samples. (A) PC1 and PC2 using ES events with average PSI values of 0.0–1.0 (all), (B) A5, (C) A3, and (D) MX, amd (E) IR. The horizontal axis represents PC1 and the vertical axis represents PC2. The percentages on the horizontal and vertical axes represent the contribution ratio of PCA.

Fig. S3
Y Deguchi

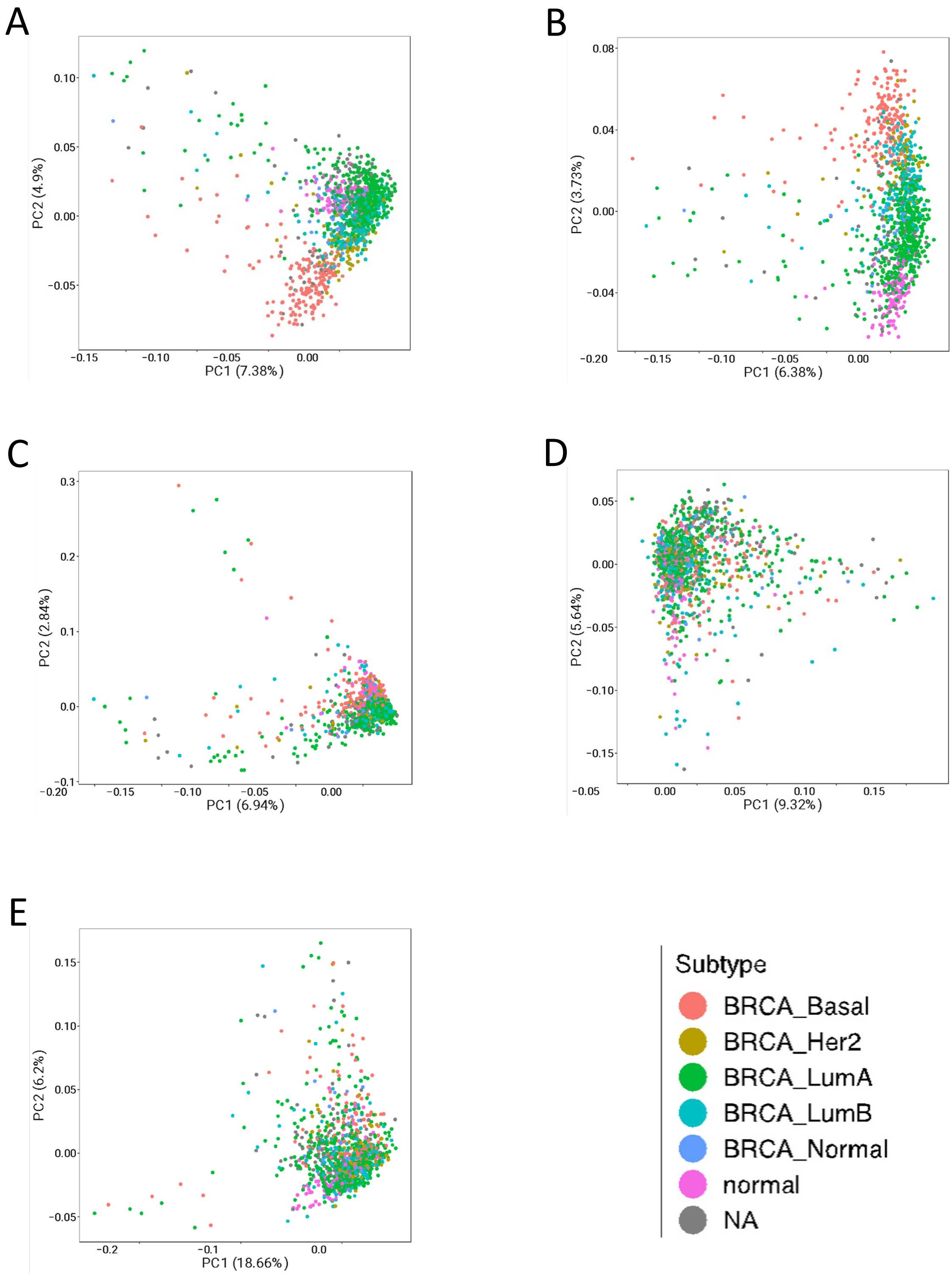

Supplementary Figure. S2 PCA results for subtype-specific AS patterns with average PSI values of 0.1–0.9

PC1 and PC2 calculated by PCA of PSI values for AS events in breast cancer samples. (A) PC1 and PC2 using ES events with average PSI values of 0.1–0.9, (B) A5, (C) A3, and (D) MX, amd (E) IR. The horizontal axis represents PC1 and the vertical axis represents PC2. The percentages on the horizontal and vertical axes represent the contribution ratio of PCA.

Fig. S4
Y Deguchi

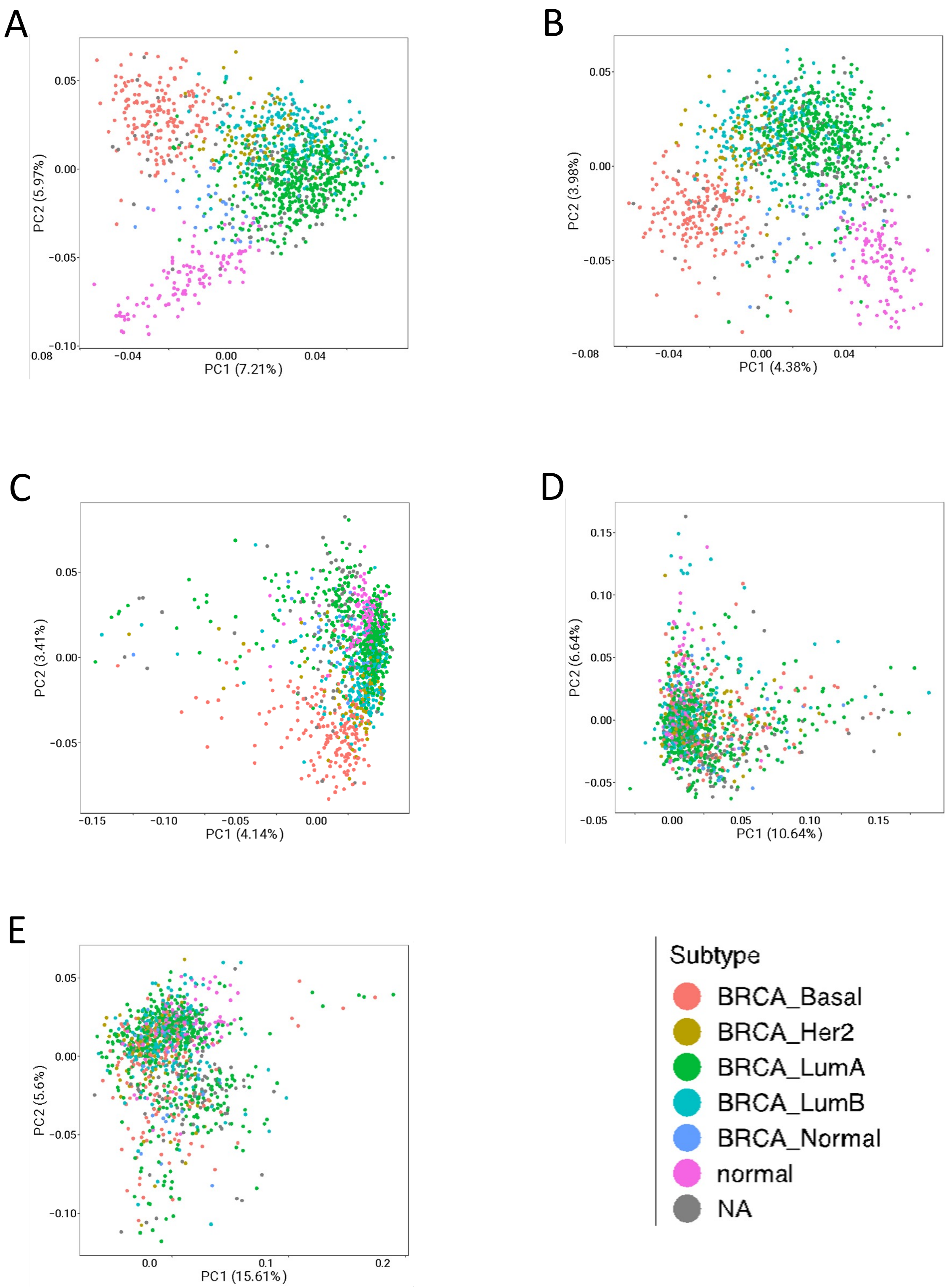

Supplementary Figure. S2 PCA results for subtype-specific AS patterns with average PSI values of 0.2–0.8

PC1 and PC2 calculated by PCA of PSI values for AS events in breast cancer samples. (A) PC1 and PC2 using ES events with average PSI values of 0.2–0.8, (B) A5, (C) A3, and (D) MX, amd (E) IR. The horizontal axis represents PC1 and the vertical axis represents PC2. The percentages on the horizontal and vertical axes represent the contribution ratio of PCA.

Fig. S5
Y Deguchi

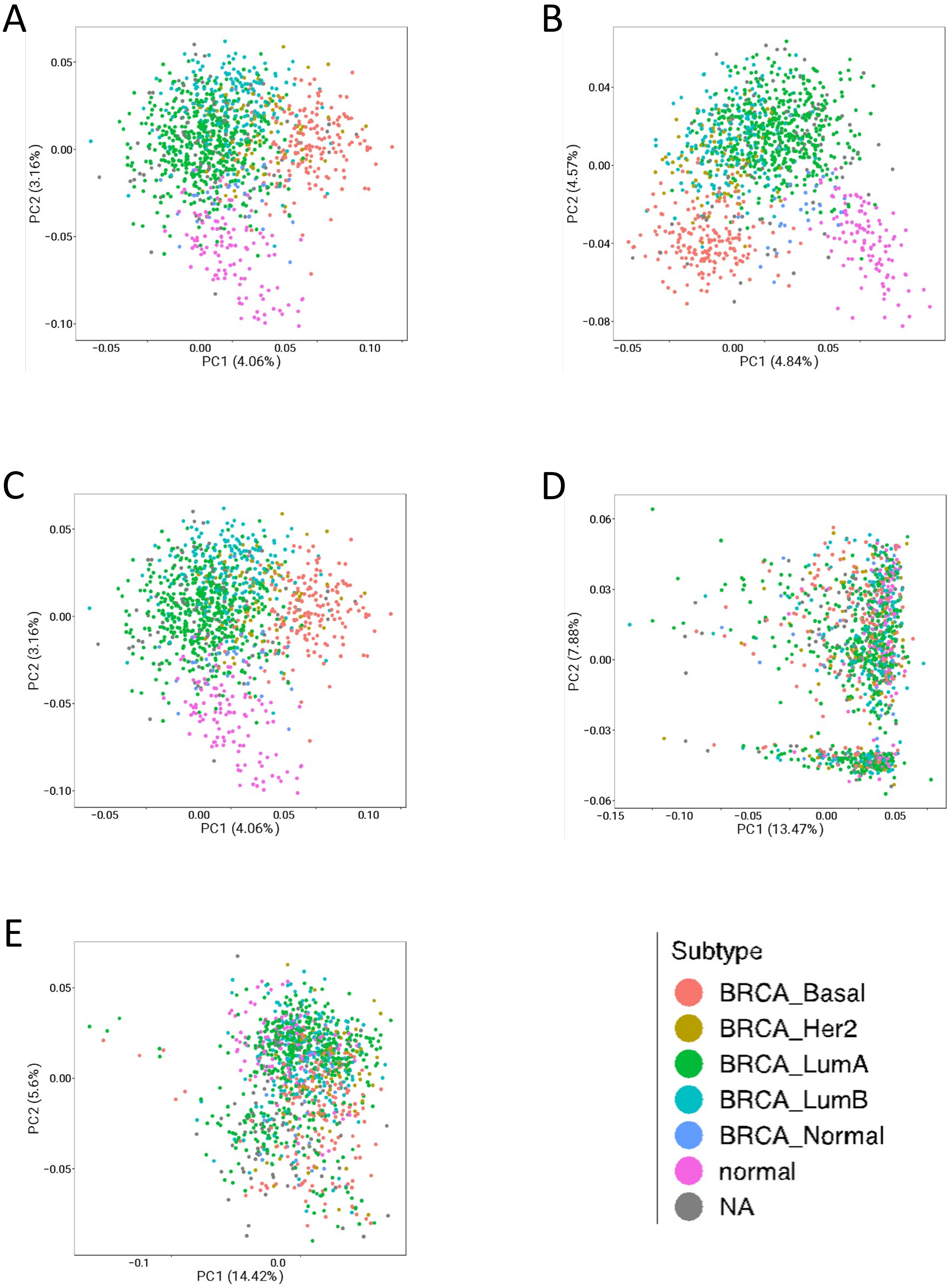

Supplementary Figure. S2 PCA results for subtype-specific AS patterns with average PSI values of 0.4–0.6

PC1 and PC2 calculated by PCA of PSI values for AS events in breast cancer samples. (A) PC1 and PC2 using ES events with average PSI values of 0.4–0.6, (B) A5, (C) A3, and (D) MX, amd (E) IR. The horizontal axis represents PC1 and the vertical axis represents PC2. The percentages on the horizontal and vertical axes represent the contribution ratio of PCA.
